# Supplementary material for: Euchromatic Histone Lysine Methyltransferase 2 Inhibition Enhances Carfilzomib Sensitivity and Overcomes Drug Resistance in Multiple Myeloma Cell Lines
Source: Cancers (Basel). 2023 Apr 7;15(8):2199. doi: 10.3390/cancers15082199 (PMC10137151; doi:10.3390/cancers15082199)
Supplement: Supplementary file 1 [file cancers-15-02199-s001.zip › cancers-2280286-supplementary.pdf]

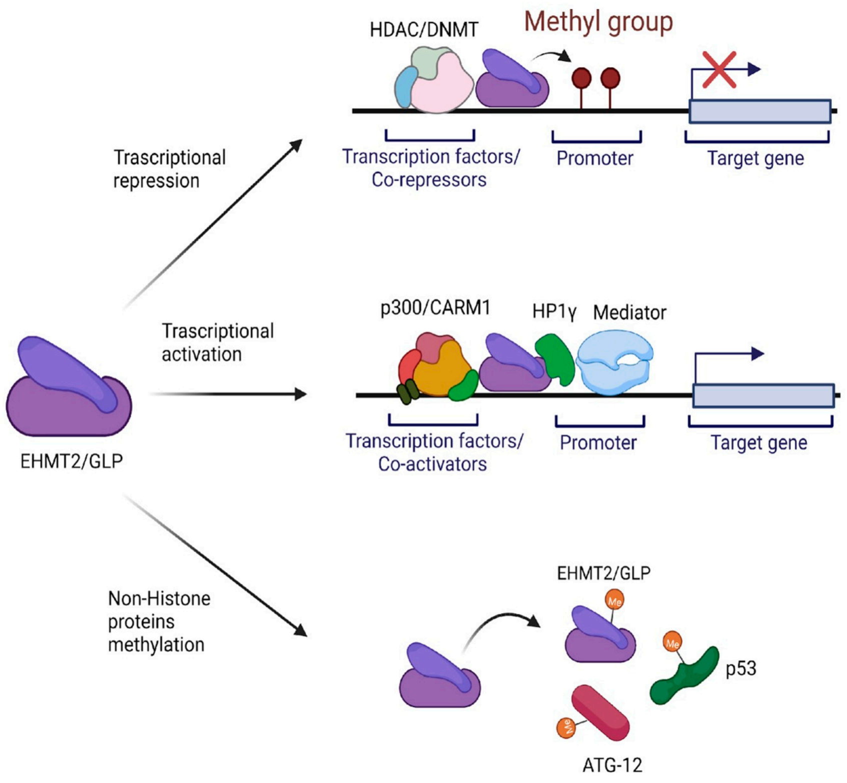

Figure S1. Roles of EHMT2 in cancer. EHMT2 interaction with co-repressors, such as HDAC, promotes histone methylation, recruitment of DNMTs and overall gene repression. Interaction with co-activators such as p300 and CARM1 stimulates recruitment of the mediators and overall gene activation. EHMT2 is capable of non-histone proteins methylation, an important post-translational modification that can regulate activity and degradation of proteins such as p53 and ATG-12.

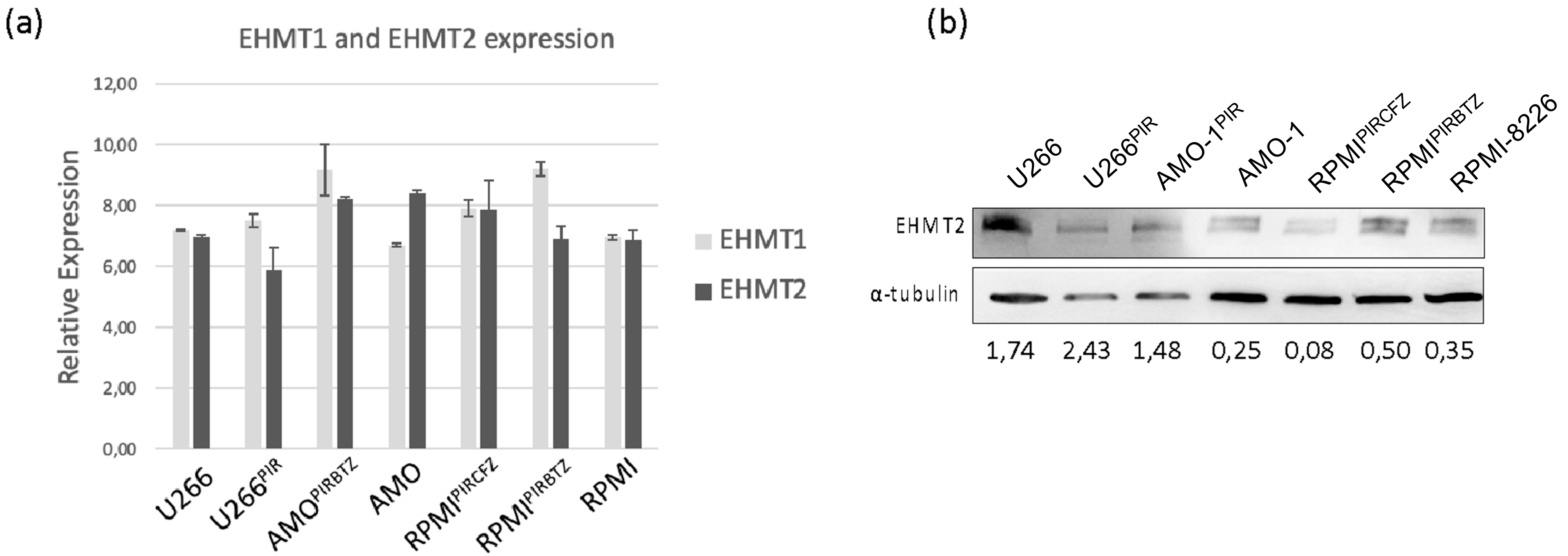

Figure S2. EHMT1/2 expression in MM cell lines. (a) RT-qPCR analysis of EHMT1 and EHMT2 expression in the indicated MM cell lines. Results are normalized to GAPDH expression. Shown are means of three technical replicates; error bars represent standard deviation (SD). (b) WB analysis of EHMT2 expression in the indicated MM cell lines and relative band quantification (EHMT2/α-Tubulin)

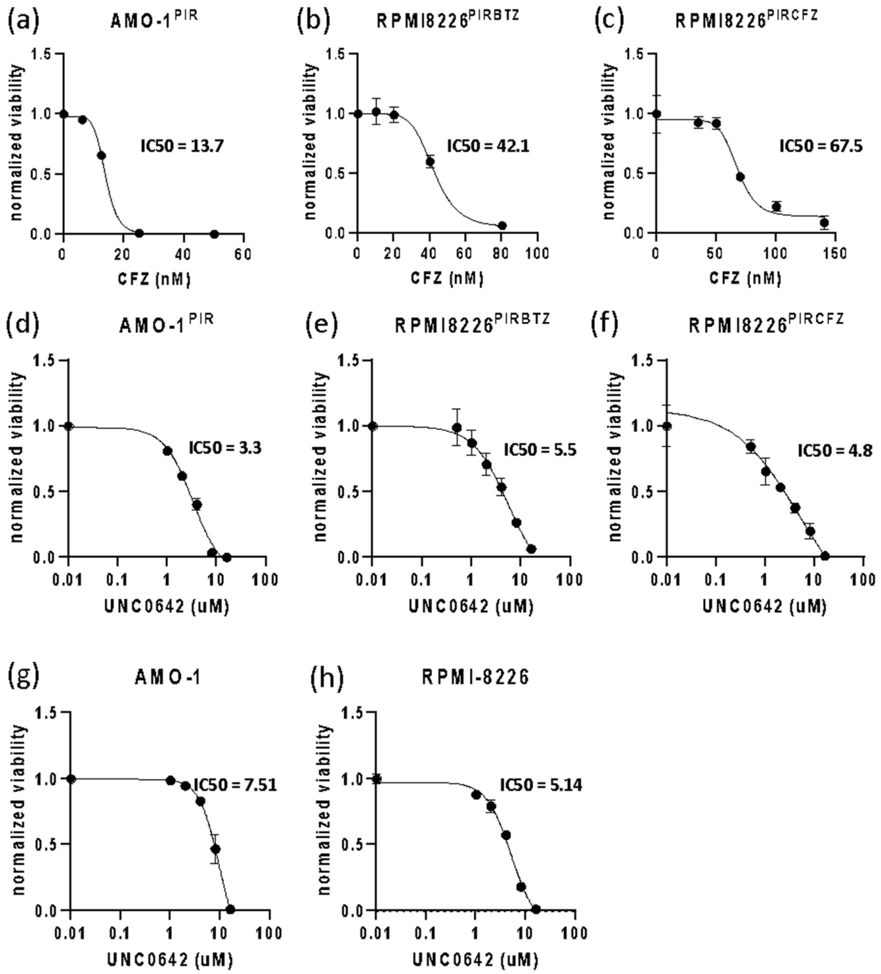

Figure S3. MM cells treated with different doses of CFZ (a-c) or UNC0642 (d-h). Viability was detected using cell growth luminescence assay 72h post treatment, error bars represent standard deviation (n=3).

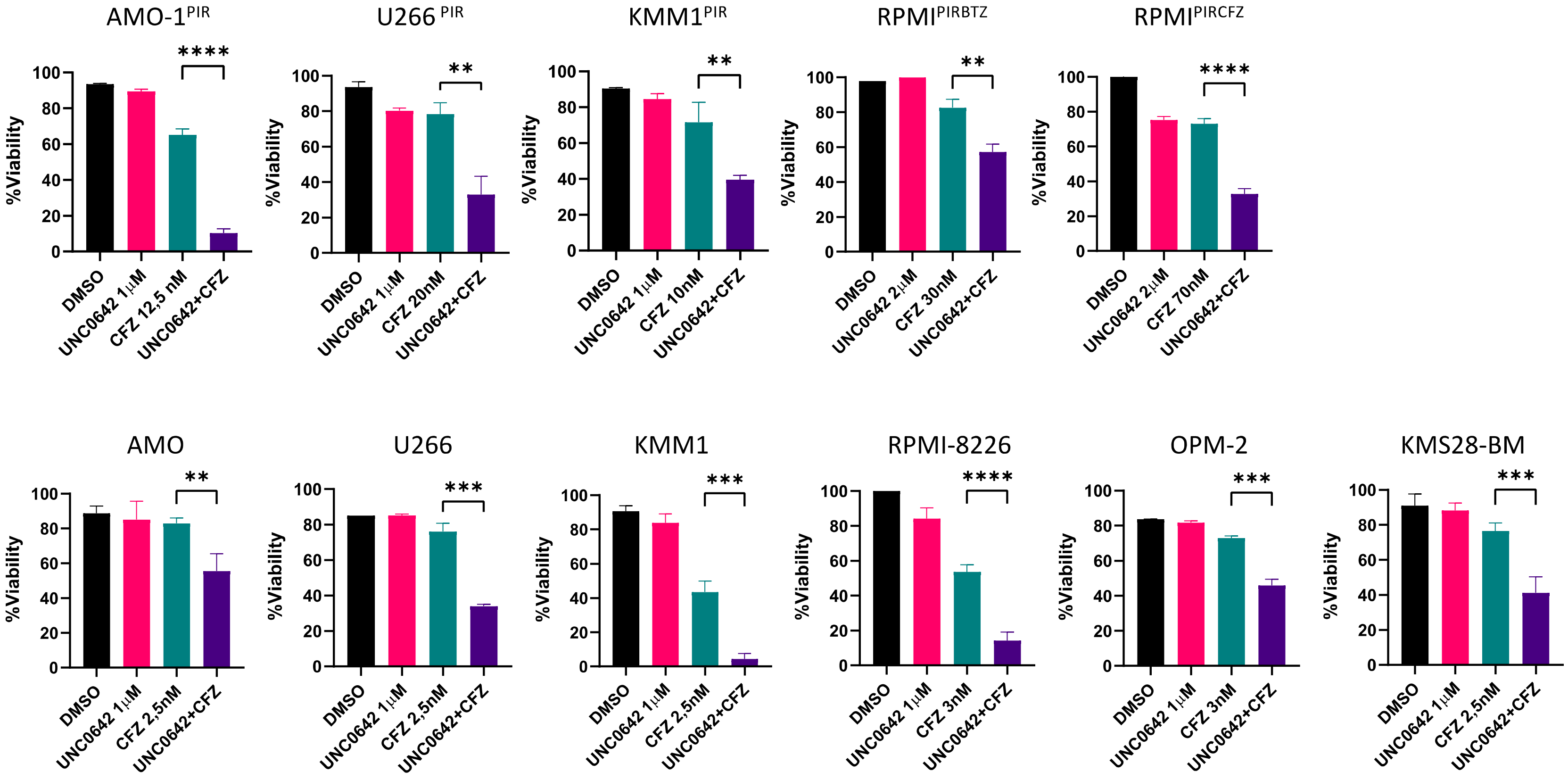

Figure S4. MM cells were treated with different dose of CFZ and UNC0642. Data are the mean of three independentexperiments, error bars represent standard deviation. Analysis performed 72h post-treatment with FACS or CellTiterGloassay (n=3).

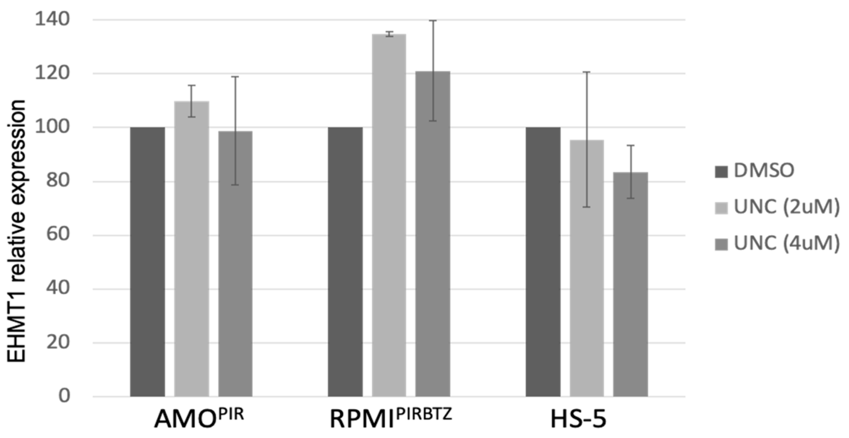

Figure S5. EHMT1 expression in AMO-1^{PIR}, RPMI^{PIRBTZ}, and HS-5, 24h post UNC0642 treatment. Data were normalized on DMSO control, error bars represent standard deviation (n=3).

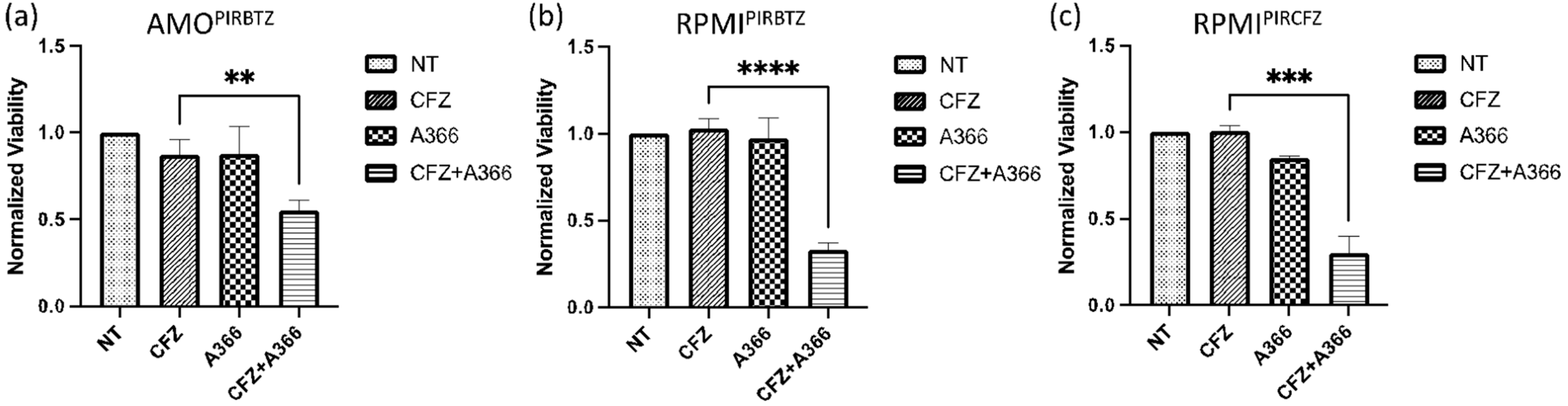

Figure S6. MM cells were treated with CFZ (RPMI^{PIRBTZ} and RPMI^{PIRCFZ} CFZ dose: 40nM, AMO^{PIRBTZ} CFZ dose: 20nM), A366 (30uM) and the combination of both. Cell Viability was assessed with cell growth luminescence assay 72h (RPMI^{PIRCFZ}) or 96h (RPMI^{PIRBTZ} and AMO^{PIRBTZ}) post treatment. Shown are means of three replicates; error bars represent standard deviation

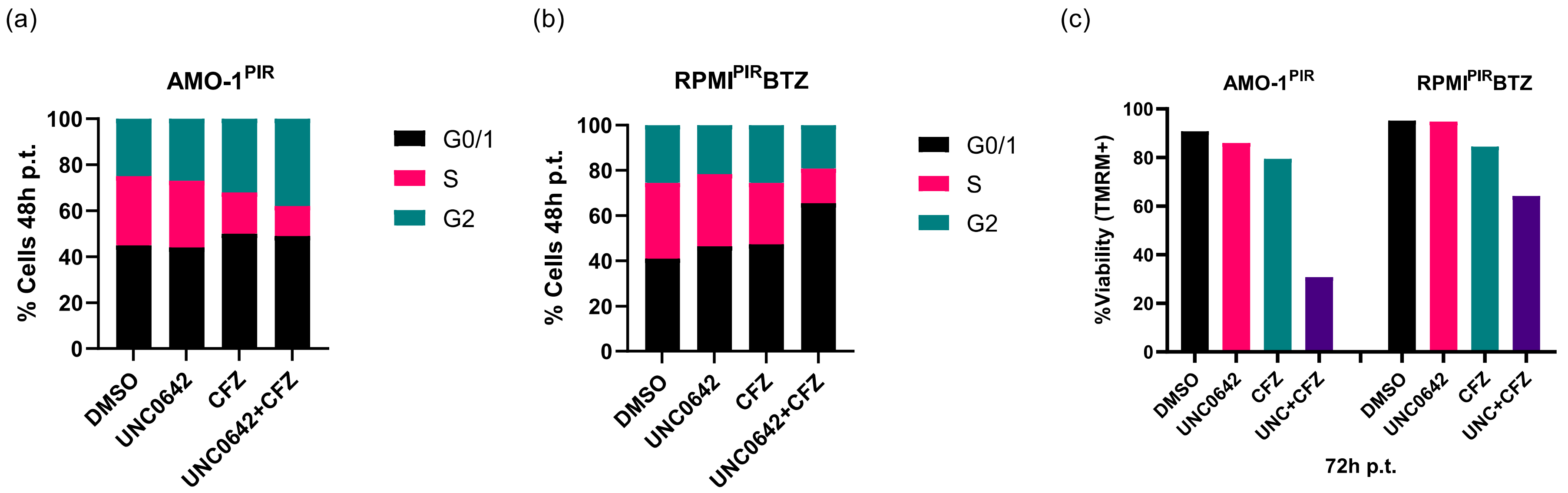

Figure S7. UNC0642/CFZ synergistic interaction is not dependent on cell cycle perturbation. Cell cycle analysis of (a) AMO-1PIR and (b) RPMIPIRBTZ cells 48h post-treatment with the indicated drugs. (c) Cell viability analysis with TMRM staining 72h post- treatment confirmed that AMO-1PIR cells are more sensitive to UNC0642/CFZ combination as compared to RPMIPIRBTZ cells. Drugs doses: 12,5nM CFZ, 1uM UNC0642 (AMO-1^{PIR}), 30nM CFZ, 2uM UNC0642 (RPMI8226^{PIRBTZ})
